# Using Transcranial Alternating Current Stimulation (tACS) to Improve Romantic Relationships Can Be a Promising Approach

**DOI:** 10.3389/fpsyg.2019.00365

**Published:** 2019-02-26

**Authors:** Shen Liu, Ru Ma, Xiaoming Liu, Chong Zhang, Yijun Chen, Chenggong Jin, Hangwei Wang, Jiangtian Cui, Xiaochu Zhang

**Affiliations:** ^1^School of Humanities and Social Science, University of Science and Technology of China, Hefei, China; ^2^Hefei National Laboratory for Physical Sciences at the Microscale and School of Life Sciences, University of Science and Technology of China, Hefei, China; ^3^School of Foreign Languages, Anhui Jianzhu University, Hefei, China; ^4^Department of Mechanical and Automation Engineering, The Chinese University of Hong Kong, Hong Kong, China; ^5^Centers for Biomedical Engineering, University of Science and Technology of China, Hefei, China; ^6^Hefei Medical Research Center on Alcohol Addiction, Anhui Mental Health Center, Hefei, China; ^7^Academy of Psychology and Behavior, Tianjin Normal University, Tianjin, China

**Keywords:** romantic relationships, transcranial alternating current stimulation, enhancement, regulation, romantic love

## Abstract

The romantic relationship refers to the specific relationship in which partners are dependent upon each other to obtain satisfactory outcomes and facilitate the pursuit of their most important needs and goals. Satisfying romantic relationships is a strong predictor of better psychological well-being, better physical health, and longer life expectancy. However, romantic relationships are not all smooth-sailing and lovers are often confronted with a variety of unavoidable issues that constantly challenge the stability of their romantic relationships. Dissatisfying romantic relationships are harmful and even destructive. Dyads of lovers engage in a variety of efforts to protect and maintain their romantic relationships based on qualitative research methods including theories- and psychological consultation-based approaches. Unfortunately, those existing approaches do not seem to effectively improve romantic relationships. Thus, it is necessary to seek an efficient approach regulating dyads of lovers in romantic relationships simultaneously. Transcranial alternating current stimulation (tACS) with advantages over existing approaches satisfies this purpose. We discuss the practicability of tACS in detail, as well as why and how tACS can be utilized to improve romantic relationships. In summary, this review firstly introduced the concept of romantic relationship and the necessity of enhancing it. Then, it discussed methods to improve romantic relationships including some existing approaches. This review next discussed the practicability of using tACS to improve romantic relationships. Finally, it shone a spotlight on potential future directions for researches aiming to improve romantic relationships.

## Introduction

Love is one of the most profound emotions known to human beings. Nothing is quite as exhilarating as falling in love. Meanwhile, nothing in the world can be fixed as frustrating, annoying, heartbreaking and unpredictable as romantic relationships. Romantic relationships can be defined as relationships in which partners are dependent upon one another to obtain satisfactory outcomes and facilitate the pursuit of their most important needs and goals (Finkel et al., 2017). A satisfying romantic relationship can strongly predict psychological well-being (Demir, 2008) and physical health (Kiecolt-Glaser and Newton, 2001). However, romantic relationships are hard to maintain fresh and exciting for long periods of time because it may fade from satisfying to dissatisfying as time goes by. Romantic relationships fail for many reasons, then leading to a great psychological anguish. Most of the lovers work consciously to master the necessary skills and take practicable measures to make them flourish. However, existing approaches to improve romantic relationships can mainly be categorized as theories- and psychological consultation-based, which does not seem to be effective because the current divorce rate in China is rising and the emotional crisis is also more and more serious (Zhang et al., 2014). Thus, it is necessary to seek a more effective way to improve and enhance romantic relationships. Especially, it is better to use a more effective approach in dyads of lovers in romantic relationships simultaneously. Non-invasive brain stimulation (NIBS) techniques, especially transcranial alternating current stimulation (tACS) seems suitable for this purpose very well.

In the present review, we firstly introduced what the romantic relationship was as well as the reason why to improve romantic relationships. Then, we discussed ways to improve romantic relationships including some existing approaches. We next discussed using tACS to improve romantic relationships and its practicability. We finally shone a spotlight on potential future directions for researches seeking to improve and enhance romantic relationships.

## Why to Improve Romantic Relationships

The romantic relationship is one of the closest and most intimate relationships that individuals have (Neyer et al., 2011) and has been demonstrated to profoundly shape individuals’ personalities. Importantly, satisfying romantic relationships are not defined by their absence of negative experiences or processes, but by their high flourishing properties such as experiences of intimacy, affection, shared fun, and perceived partner responsiveness (Gottman et al., 1998). Satisfying romantic relationships strongly influence individuals’ behaviors and development (Kuo et al., 2017) and are critically important for many reasons. For example, satisfying romantic relationships are responsible for enhanced psychological well-being (Medvedev and Landhuis, 2018) and favorable physical health (Holt-Lunstad et al., 2010). Engaging in one’s first romantic relationship was linked to decreases in neuroticism and shyness, and increases in extraversion, conscientiousness, and self-esteem (Neyer and Lehnart, 2007; Lehnart et al., 2010). In addition, further satisfying relationship-related experiences such as moving in with one’s partner, marrying was shown to contribute to personality development (Jonkmann et al., 2014). Moreover, satisfying experiences within relationships such as feeling close and having conflicts predicted both changes in self-esteem (Mund et al., 2015) and negative emotionality in young adulthood (Robins et al., 2002). Therefore, it’s critical for dyads of lovers to maintain stable romantic relationships.

However, romantic relationships are not all smooth-sailing and dyads of lovers often confront a variety of unavoidable issues that constantly challenge the stability of their romantic relationships. Some stable romantic relationships may be hurt or even dissatisfying, which may be destructive and even harmful (Specht et al., 2011). The following are some possible reasons leading to dissatisfying romantic relationships. Good communication, one of the characteristics shared by happy dyads of lovers, is essential to romantic relationships (Rodriguez et al., 2016). However, communication difficulty is among the top reasons that lovers report as having caused their romantic relationships dissatisfaction or even destruction (Miano et al., 2017). Even worse, with the expansion of communication technologies over the years, non-face-to-face communication has become increasingly popular and important (Luo and Tuney, 2015), while it unfortunately, becomes a harmful factor to romantic relationships (Luo, 2014; Hampton, 2016). Mindfulness, although as a positive trait, it may also be a destructive factor leading to dissatisfying romantic relationships. Low mindfulness was associated with decreased relationship satisfaction (Barnes et al., 2007), and reduced satisfaction with interpersonal relationships more broadly (Pepping et al., 2014). Gratitude, as well as being a positive trait, may also lead to dissatisfying romantic relationships. For example, self-gratitude is associated with higher romantic relationship satisfaction while indebtedness is not associated with relationship satisfaction (Algoe et al., 2010). While individuals’ felt and expressed gratitude both significantly related to their own marital satisfaction, only their felt gratitude but not their expressed gratitude predicted the spouse’s satisfaction (Gordon et al., 2011). Positive emotion, another trait, may also lead to dissatisfying romantic relationships. For example, previous researches on newlywed couples indicated that couples with less affection, less interests in their partners, or displaying more anger, complaint or criticism in their interactions were reported with lower satisfaction. Those couples even reported poorer physical health, compared to couples who performed more positive affect including agreement and humor in their interactions (Gottman and Levenson, 1992; Carrere and Gottman, 1999). Other studies showed that a lack of positive emotion expression was a robust predictor of dissatisfying and unstable romantic relationships (Gable et al., 2006) and would destroy romantic relationships significantly (Mirgain and Cordova, 2007).

Therefore, satisfying romantic relationships have numerous benefits while dissatisfying romantic relationships bring many bad consequences. Whether from pursuing benefits of satisfying romantic relationships or from avoiding harm of dissatisfying romantic relationships, it is necessary to engage in a variety of efforts to protect and maintain romantic relationships. The following part mainly introduced and discussed existing approaches related to improve romantic relationships.

## How to Improve Romantic Relationships

Ordinary people, psychologists and therapists are plagued with the questions of how to improve romantic relationships. Existing approaches to improve romantic relationships are mainly qualitative research methods and can be categorized as theories- and psychological consultation-based.

There are two representative theories corresponding to improving romantic relationships. One is the Relational Maintenance Theory, which describes that there are some strategies used by lovers to improve their romantic relationships (Canary and Stafford, 1992). Strategies described in this theory are categorized some behaviors, such as positivity (expressing positive affect to partners), openness (disclosing one’s wants or needs from romantic relationships), assurances (expressing one’s commitment to partners in reassurance) and so on (Rus and Tiemensma, 2017). For example, Stafford et al. (2000) adapted the commonly used Relational Maintenance Strategies Measure to Facebook behaviors and found that both Facebook positivity (e.g., sending cheerful messages to a partner) was associated with relationship satisfaction. The other is the Uncertainty Reduction Theory, which states that partners use communicative behaviors to decrease uncertainty about their romantic relationships (Berger and Calabrese, 1975). Knobloch and Solomon (1999) identified four distinct types of relational uncertainty including behavioral uncertainty, mutuality uncertainty, future uncertainty and definitional uncertainty. Stewart et al. (2014) further indicated that specific types of uncertainty predicted both monitoring and relationship maintenance behaviors.

Psychological consultants always put forward some principles contributing to improving romantic relationships. For example, Finkel et al. (2017) conclude that there are fourteen core principles related to improving romantic relationships, such as maintenance, integration and so on. Usually, psychological consultants suggest some ways for improving romantic relationships built on the above-mentioned principles. For example, as for the maintenance, partners in romantic relationships exhibit cognition and behaviors that promote their relationship’s persistence over time, even if it involves self-deceptive biases (Simpson and Rholes, 2017). Psychological consultants also indicated that the romantic relationship-maintenance mechanisms contributed to improving romantic relationships. One of the most robust predictors of the tendency to enact romantic relationship-maintenance mechanisms is romantic relationship commitment, which emerges from feelings of satisfaction and investment in the romantic relationship and from the belief that the alternatives to involvement in the romantic relationship are less desirable (Le and Agnew, 2003).

However, those existing approaches do not seem effectively to improve romantic relationships. Theories- or psychological consultation-based approaches are qualitative research methods and are all put forward some theoretical frames or principles on the macro with a lack of empirical evidence to be validated. They don’t put the reasons behind the behavior (e.g., brain activity) into consideration. Even worse, although some of these methods have been put into use for a long time, the current divorce rate in China is rising and the emotional crisis is also more and more serious (Zhang et al., 2014). Therefore, it is necessary to find a more efficient approach. Especially, it is better to use the more efficient approach simultaneously in dyads of lovers in romantic relationships. NIBS techniques, especially tACS seems well suited for this purpose.

## Using Transcranial Alternating Current Stimulation (tACS) to Improve Romantic Relationships

The tACS technique, as a valuable tool to modulate activities of cerebral areas in humans, has been tested both in healthy (Arendsen et al., 2018) and in several clinical populations suffering from various conditions such as Parkinson’s disease (Brittain et al., 2013; Mellin et al., 2018). The major parameters determining the effect of tACS are frequency (or frequency range), intensity and phase (phase relationship of two electrode pairs). During the half cycle of an AC (tACS) oscillation, one electrode acts as the anode while the other acts as the cathode. During the other half cycle, the pattern reverses, i.e., the former anode now serves as a cathode and the former serves as an anode. The current strength increases and decreases following a half sine wave (Carvalho et al., 2018).

A growing body of studies has shown that tACS is able to modify cortical excitability and activity as well as behavioral performance in various domains, such as memory or motor function (Antal and Paulus, 2013; Herrmann et al., 2013). Applying tACS at frequencies in the EEG range entrains neuronal networks at the applied frequency (Antal and Paulus, 2013; Herrmann et al., 2013). For example, tACS applied at alpha and high gamma frequencies over the somatosensory cortex elicits tactile sensations in a frequency-dependent manner (Feurra et al., 2011). Furthermore, such targeting of specific EEG frequency ranges has been demonstrated to enhance performance in the associated cognitive domains. For example, tACS in the alpha range over visual cortex improved performance in a visual conjunction search (Muller et al., 2015) and somatosensory alpha tACS could reduce pain experience (Arendsen et al., 2018). Thus, tACS makes it possible to study causal relationships between specific oscillations and corresponding cognitive functions. Further, its use can offer promising new pathways for therapeutic applications to treat neurological or psychiatric disorders associated with dysfunctional neuronal oscillations (Kasten et al., 2018). For example, Brittain et al. (2013) utilized tACS over the motor cortex to induce phase cancelation of the rest tremor rhythm. Mellin et al. (2018) used tACS to improve persistent auditory hallucinations in patients with schizophrenia. Improving romantic relationships is one of the contents of psychiatric research and we believe that utilizing tACS can improve romantic relationships.

Except modulating brain oscillation at only one brain region, tACS can also be used to modulate coherence between two spatially distant regions, which are belonging to one functional network. For instance, Polania et al. (2012) utilized tACS to exogenously induce synchronized or desynchronized fronto-parietal theta coupling with the application of 6 Hz (theta band) tACS over the left prefrontal and parietal cortices with a relative 0° (in-phase stimulation) or 180° (anti-phase stimulation) phase difference. FMRI during tACS also supported that fronto-parietal theta in-phase stimulation successfully increased neural activity of the fronto-parietal network while increased deactivation was found in nodes of the default mode network during anti-phase stimulation (Violante et al., 2017). In-phase stimulation could also facilitate interhemispheric functional connectivity and increase motion perception compared with anti-phase stimulation when applied in 40 Hz (gamma band) at bilateral parieto-occipital areas (Helfrich et al., 2014).

Just as the connectivity of two brain regions in one brain network can be modulate by tACS, brain activity of two persons in one social network (e.g., a dyad of lover) may also be regulate by tACS at the same time. While theoretically speaking, dyads of lovers in romantic relationships should be simultaneously regulated to improve their romantic relationships. In the present review, we adapted the logic of the Polania et al. (2012) to inter-brain synchronized networks. Instead of modulating the oscillatory phase between stimulation electrodes on one head and thus boosting or disrupting intra-brain synchronized oscillations, tACS is applied simultaneously to two individuals (hyper-tACS) to modulate frequency and phase between stimulation electrodes on two heads. Using tACS to regulate dyads of lovers in romantic relationships simultaneously mainly relies on specific neuronal oscillations. Increasing amount of studies found that dyads of lovers showed some specific inter-brain synchronizations. For example, lover dyads compared with friend and stranger dyads behaved better in cooperation task and showed increased cross-brain coherence in right superior frontal cortex (Pan et al., 2017). The inter-brain synchronization was found significant in temporal-parietal gamma activity during naturalistic social interactions (e.g., looking at each other) between dyads of lovers (Kinreich et al., 2017). Similarly, Goldstein et al. (2018) demonstrated that the mechanism underlying touch-related analgesia between dyads of lovers might be the inter-brain coupling in alpha-mu (8–12 Hz) frequency band. One of our ongoing studies also found that dyads of lovers showed significantly higher inter-brain synchrony at specific frequency bands during affective interaction. In this manner, we hope to boost inter-brain synchronized oscillations of lover dyads and examine the effect of this manipulation on the degree of behavioral synchronization, namely the enhancement of romantic relationships. We hypothesize that if inter-brain oscillatory couplings are indeed constitutive for the enhancement of romantic relationships, experimental modulation of inter-brain oscillatory synchronization might affect the degree of romantic relationships. Fortunately, the similar idea has been tried by some researchers. For example, Szymanski et al. (2017) applied tACS simultaneously to two individuals who were asked to drum in synchrony at a set pace and found that same-phase-same-frequency stimulation would improve interpersonal action coordination. Novembre et al. (2017) applied hyper-tACS during a dyadic finger tapping task in left centroparietal areas at the beta frequency (13–30 Hz) to interfere with synchronization processes in motor regions specifically and indeed report facilitation of early inter-personal action synchronization in a same-phase-same-frequency relative to different-phase-same-frequency. Considering these findings, we further hypothesized that same-phase-same-frequency hyper-tACS would improve dyadic synchronization of lovers in romantic relationships, while different-phase-different-frequency hyper-tACS would weaken dyadic synchronization of lovers in romantic relationships. In order to investigate the continuing effects of tACS, some follow-ups are needed to be performed. The follow-ups will be performed after one week, one month, three months and six months of the tACS. Dyads of lovers are required to perform the same cognitive tasks and complete the same questionnaires or scales as before tACS in the follow-ups.

Built on the above-mentioned, we could gather that tACS can identify causal relations between brain physiology and behavior, and thus it is a possible means to complement current knowledge in this field. However, there is yet no study using tACS to regulate dyads of lovers in romantic relationships simultaneously. But it can be assumed that using tACS to regulate both short-term and long-term romantic relationships (especially for dyads of lovers in dissatisfying romantic relationships) is convincing and promising, as it can directly modulate brain activity underlying behavior of lover dyads compared with theories- and psychological consultation-based methods.

## Future Directions

As the tACS technique is used for exploring basic aspects of human brain physiology, cognitive functions or suitability, it has experienced significant growth in the past few years (Naro et al., 2016; Alexander et al., 2018). For the purpose of achieving the application of tACS in improving romantic relationships to a great extent, the following possible future directions are worth considering.

On the one hand, the tACS technique should be utilized in combination with neuroimaging techniques, such as fMRI and EEG (electroencephalograph), to closely identify underlying functional brain networks relevant to romantic relationships. For example, tACS can be utilized to manipulate neuronal oscillations while whether these manipulations of neuronal oscillations can also affect oxygen or metabolic consumption is unknown. Although several studies have already shown that specific oscillatory brain activity is linked to certain patterns of brain activation (Tyvaert et al., 2008; Michels et al., 2010), studies direct linking tACS to fMRI are few. Using tACS to regulate dyads of lovers in romantic relationships simultaneously mainly relies on specific neuronal oscillations while this method cannot accurately locate brain regions related to romantic relationships. However, fMRI can record the signal from all regions of the brain and can non-invasively record brain signals without the risks of ionizing radiation inherent in other scanning methods, such as PET (Positron Emission Tomography) scans. Therefore, it can be presumed that combining tACS with neuroimaging techniques can better improve romantic relationships.

On the other hand, tACS has been proved to be a promising therapeutic intervention for diverse psychiatric and neurological conditions. This technique has appealing characteristics, such as being non-invasive, well-tolerated, and so far, absent of any serious adverse effects (Nguyen et al., 2018). NIBS have been used as a therapeutic intervention while only tDCS is increasingly used for psychiatric disorders treatment (Moffa et al., 2018). Although the main advantages of tDCS among all forms of NIBS are its low cost, portability, and potential for home-administered use, ease of use, and absence of severe adverse effects, which may increase the adherence of psychiatric patients to the treatment, tDCS cannot achieve the goal of simultaneously regulating dyads of participants. From this point of view, tACS should be widely used as a therapeutic intervention especially for dyads of participants.

In conclusion, the romantic relationship has proven to be an increasingly interesting topic to neuroscientists and social psychologists, but there is still much to be further discovered in order to maintain, enhance and improve the romantic relationships. tACS seems well suited for this purpose, which can be used to modulate intra-brain synchronized networks of dyads of lovers.

## Author Contributions

SL and XZ conceived and wrote the frame design. SL, RM, CZ, YC, CJ, HW, JC, and XZ wrote the manuscript. All authors revised the manuscript.

## Conflict of Interest Statement

The authors declare that the research was conducted in the absence of any commercial or financial relationships that could be construed as a potential conflict of interest.
